# Carotid Ultrasound Screening Programs in Rural Communities: A Systematic Review

**DOI:** 10.3390/jpm11090897

**Published:** 2021-09-08

**Authors:** Marjana Petrova, Hosen Kiat, Alex Gavino, Craig S. McLachlan

**Affiliations:** 1 Centre for Healthy Futures, Health Faculty, Torrens University, 5/235 Pyrmont St., Pyrmont, NSW 2009, Australia; hosen.kiat@chi.org.au (H.K.); alex.gavino@torrens.edu.au (A.G.); craig.mclachlan@torrens.edu.au (C.S.M.); 2 Faculty of Medicine, Health and Human Sciences, Macquarie University, Level 3, 75 Talavera Rd., Sydney, NSW 2109, Australia; 3 School of Rural Medicine, University of New South Wales, 559 East St., Albury, NSW 2640, Australia

**Keywords:** carotid ultrasound, carotid atherosclerosis, rural screening, carotid plaque

## Abstract

Carotid atherosclerosis assessments inform about stroke and cardiovascular risk. It is known that stroke and cardiovascular disease (CVD) prevalence is higher in rural communities than in urban communities. We aimed to conduct a systematic review of rural carotid ultrasound screening programs to define carotid atherosclerosis using traditional and emerging imaging biomarkers, prevalence, and risk factors. We searched Ovid/MEDLINE, Ovid/EMBASE, SCOPUS and CINAHL from inception to 3 April 2020 for rural population studies that utilized carotid ultrasound screening for adults ≥40 years of age and free of known cerebrovascular disease. Studies were included if participants received a bilateral ultrasound scanning of the carotid arteries and reported at least one marker of carotid atherosclerosis pathology. A random effect meta-analyses calculated the estimated prevalence of carotid plaque. In total, 22/3461 articles that met all of the inclusion criteria were included. Studies reported increased intima media thickness (IMT), carotid plaque presence and carotid artery stenosis. There were no studies reporting on novel imaging markers, such as carotid stiffness, carotid plaque morphology or neovascularization. The overall random effect pooled prevalence of carotid plaque was 34.1% (95% CI, 33.6–35.0); the prevalence of increased IMT was 11.2–41.5%, and the prevalence of carotid artery stenosis was 0.4–16.0%. There is an absence of data necessary to understand the carotid atherosclerosis prevalence across global rural populations. Represented studies have focused on East Asian countries where a high burden of rural carotid artery disease has been reported. There is no rural evidence to guide the use of novel ultrasound carotid biomarkers such as stiffness or neovascularization.

## 1. Introduction

The evaluation of modifiable environmental risk factors is the traditional approach to predicting those that should be treated to prevent cardiovascular disease (CVD) outcomes [1]. However, in many cases, the subclinical pathology of cardiovascular disease is present in individuals with no overt risk factors. For example, subclinical atherothrombosis and/or atherosclerosis is frequently related to premature death through acute myocardial infarction; stroke; or acute exacerbation of chronic diseases, such as renal and/or heart failure [2,3]. To assess the risk of subclinical atherosclerosis, a range of non-invasive carotid imaging markers are available, which provide a robust method for diagnosing subclinical and asymptomatic atherosclerotic disease [4]. Specifically, ultrasonography is an accessible imaging modality that can be used to predict cardiovascular outcomes via the assessment of detectable carotid atherosclerotic plaque, increased intima media thickness (IMT) and carotid artery stenosis [5,6,7]. Besides these traditional ultrasound determinants, studies have demonstrated emerging ultrasound biomarkers (e.g., carotid stiffness, carotid plaque morphology and neovascularization) as risk factors for stroke and future cardiovascular outcomes [8,9]. The stiffening of the carotid artery is considered an important element of the pathogenesis of cerebrovascular disease and has also been identified as a potential independent risk factor for stroke, vascular dementia, and depression [10]. Moreover, increased carotid stiffness has been associated with CVD events and all-cause mortality [11,12]. It is of interest that The European Society for Vascular Surgery and the European Society of Cardiology have both developed a consensus recommendation for the evaluation of carotid plaque morphology in patients with asymptomatic carotid artery disease [13].

Carotid artery screening for stroke and CVD prevention in the general population is controversial. Current guidelines do not recommend screening for carotid artery disease in asymptomatic communities. Guideline evidence has been reliant on the harms and benefits of carotid endarterectomy and stenting [14]. As atherosclerosis is a chronic, widespread, and progressive disease [7,15,16,17], early detection of carotid atherosclerosis and associated carotid pathology could be beneficial not only for risk stratification but also for obtaining a better understanding of the general cardiovascular health and risk factors of asymptomatic patients. Rural residence has been associated with a higher burden of stroke, ischemic heart disease and associated risk factors in population-based studies, as well as a lack of specialized health services when compared with urban communities [18,19,20]. To the best of our knowledge, a review of community-based rural studies for carotid screening has not been previously undertaken. A clear understanding of the burden of carotid atherosclerosis and an overview of screening programs in rural communities is crucial to strategic planning for the prevention of cardiovascular disease and the improvement of rural health outcomes. Our primary aim is to provide a comprehensive systematic review of screening programs using carotid ultrasound, ultrasound imaging biomarkers and the associated risk factors in those aged 40 and above who are free of symptomatic carotid artery disease. Age 40 was chosen as the cutoff for the burden of environmental risk factors present prior to the age of 40 reflects subclinical atherosclerotic burden in middle age (40–50 years) [21]. The secondary aim of this study is to determine whether it is possible to estimate the prevalence of carotid atherosclerosis in rural communities using the limited published data.

## 2. Materials and Methods

### 2.1. Search Strategy

In this systematic review, multiple databases were searched including Ovid/MEDLINE, Ovid/EMBASE, SCOPUS, and Cumulative Index to Nursing and Allied Health Literature (CINAHL). The searches were conducted from their inception to 3 April 2020. The following keywords for the search strategy were used: (carotid) AND (ultrasound OR sonography OR ultrasonography) AND (rural OR regional OR remote). Details of the electronic search strategy can be found in the Supplementary Material, Table S1. Additionally, articles’ reference lists and Google search were also screened to retrieve additional articles.

### 2.2. Study Eligibility

Titles and abstracts were reviewed to identify potential studies that aligned with the predefined inclusion criteria: (i) general studies that were conducted in rural, remote, or regional settings in adult populations ≥ 40 years of age who were free of symptomatic carotid artery disease; (ii) studies that included participants who had undergone bilateral carotid ultrasonography scanning and that had reported on at least one ultrasound determinant of carotid pathology; (iii) studies with either a cross-sectional or prospective cohort by design, and articles written in English; (iv) studies conducted in a mixed population that had their results presented separately for rural and urban areas; and (v) studies that reported/analyzed outcomes of interest but did not report on their prevalence.

In the instances where the rurality of the study setting was not explicitly stated by the authors, we used the Degree of Urbanization recommendations developed by the UN Statistical Commission [22] to determine if the study was conducted in a rural or an urban location.

Multiple reports from the same study population where different outcomes, sub- analyses or overlapping variable outcomes were presented were not excluded from the review, but only the most recent publication from each one was considered in the analyses to estimate risk factors and prevalence.

Conversely, we excluded studies that (i) included patients with a history of cerebrovascular diseases such as stroke and transient ischemic attack; (ii) had a history of carotid endarterectomy (CEA) or stenting; (iii) were conducted among selected healthy volunteers to determine the normal parameters of IMT; (iv) were case-control studies, retrospective studies (medical records, hospital database, registries etc.) or randomized controlled trials; or (v) were confined to urban areas. 

### 2.3. Outcomes

The primary outcome was carotid artery pathology (and related ultrasound determinants) and/or stiffness detected by ultrasound. In studies where the prevalence of carotid artery plaque was present, the pooled prevalence was calculated as a secondary outcome.

### 2.4. Data Extraction and Analysis

The initial review of the manuscripts (first pass) included: author’s name, journal, year of publication, country, duration, study setting (rural or rural/urban), study design, reason for screening, number of participants, age range, mean age, gender distribution, race, inclusion and exclusion criteria, ultrasound technique, ultrasound parameters, outcomes, and results. The quality of the included studies was determined based on the Strengthening the Reporting of Observational Studies in Epidemiology statement (STROBE) checklist across five modules: sample population, sample size, participation rate, outcome assessment, and analytical methods. Each study received a score result on a scale of ten (Supplementary Material, Table S2). The scoring scale and the assessment of each module were adopted from Song et al. [23].

The prevalence of carotid plaque in rural populations was pooled across studies with the use of a random-effect model, which allows for a heterogeneity of effect among the studies. A range of prevalence was provided for increased IMT and carotid artery stenosis due to the high heterogeneity of the included studies and the heterogeneous criteria and definitions of the abovementioned outcomes. Risk factors among the studies were investigated with a narrative review, due to insufficient data for a robust meta-analysis.

Meta-analyses for prevalence were conducted with Comprehensive Meta Analyses (CMA) software (version 3·0).

## 3. Results

**Figure 1:**
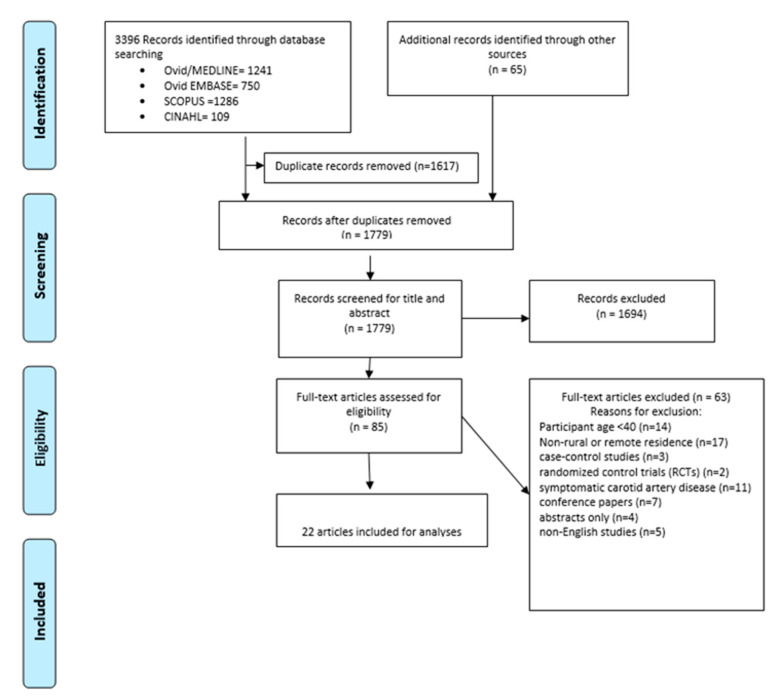
Study selection.

After removing the duplicates from 3451 records, 1776 titles and abstracts were screened in compliance with our predefined inclusion and exclusion criteria. In total, 85 full text articles were reviewed and 22 were included in the systematic review analysis (Figure 1). The included articles were published between 1999 and 2020 and covered five countries across four continents as follows: Japan = 5; Ecuador = 3, USA = 1; Korea = 1, and China = 12. All of the included articles had a quality score of 6 and above, apart from one study that had a score of 5 [24].

All of the included studies were cross-sectional and general population based. Participants were recruited from cardiovascular surveys, general health check-ups and community screening programs. Only one study from the USA represented a targeted vascular screening program [25]. A total of 11 articles reported on IMT [24,26,27,28,29,30,31,32,33,34,35], 11 reported on carotid plaque [24,25,31,32,36,37,38,39,40,41,42] and five reported on carotid stenosis [24,25,33,35,43]. Our search strategy did not yield any articles reporting on carotid stiffness or other carotid ultrasound parameters. A total of 19 articles (86%) included in this review were published in the last decade (2010–2020), of which 20 were conducted exclusively in rural settings and two were conducted in a mixed rural/urban setting [32,35]. The detailed characteristics of the included studies are shown in Table 1.

### 3.1. Rural Studies

There was a total of seven reports from the Tianjin Brain Study, a population-based study on stroke mortality and incidence in 18 rural villages in China. Five of these studies were sub-studies that analysed the relationship between carotid IMT and carotid plaques, and the following: hypertension [35,45], glucose levels [29,30] or body mass index (BMI) [41]. The Atahualpa project was a population-based survey designed to investigate the presence of risk factors for CVD and stroke in rural Ecuador [46]. Two of the Atahualpa sub-studies analysed the correlation between cognitive performance, sleep quality and IMT [26,34], while another explored the relationship between the presence of an earlobe crease and atherosclerotic plaque [27]. The Hisayama Study from Japan examined the association between chronic obstructive pulmonary disease (COPD), carotid plaque and IMT [44].

Other studies in the review examined the following relationships: ankle-brachial index (ABI) and IMT [37], homocysteine and increased IMT and carotid plaque [24], and IMT and abdominal calcification [44].

### 3.2. Prevalence of Increased IMT

The prevalence of increased IMT was reported in five studies, which were conducted in three countries: Japan, China and Ecuador [24,31,32,33,34]. The lowest prevalence of increased IMT (11.2%) was reported in the Xing Long Zhuang community in China, while the highest (41.5%) was reported in Taizhou City, Jiangsu Province in China. The criteria for increased IMT varied among studies, ranging from IMT ≥ 0·9 mm to IMT ≥ 1·8 mm (Table 2).

### 3.3. Prevalence of Carotid Plaque 

**Figure 2:**
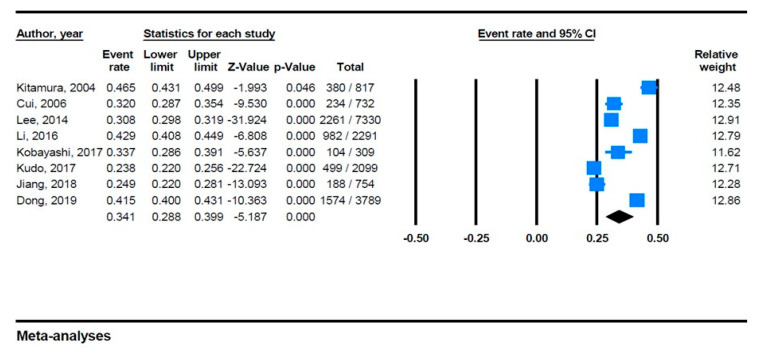
Meta-analyses of carotid plaque prevalence.

Through eight studies across China, Japan, and Korea, we obtained data from 18,879 participants. The sample population size of these studies ranged from 309 to 7330, with men representing 46.2%. The age cutoffs for the studies were 40, 45, 55 and 60 years. The lowest prevalence (23.7%) of carotid plaque was reported in a population study based in Hisayama, Japan (The Hisayama Study) [36], and the highest prevalence (46.5%) was reported among the rural portion of a mixed population cardiovascular survey also in Japan [32]. The prevalence of carotid plaque with a pooled random effect estimated 34.1% (95% CI 28.8–39.9%) (Figure 2.) Examination of the funnel plot (not presented) showed no asymmetry for studies on the prevalence of carotid plaque (Egger’s test *p* = 0·485). The accepted criteria for the presence of carotid plaque in all included studies was IMT ≥ 1·5 mm, which corresponds to the Manheim IMT and carotid plaque consensus [47].

The largest study on the prevalence of carotid atherosclerosis that we reviewed was excluded from the meta-analyses due to the large sample size. This study was a part of the China National Stroke Prevention Project, comprising 84,880 participants from 31 Provinces in mainland China, of which 44,220 (51.2%) were living in rural communities [35]. It was also noted that the composite prevalence of carotid atherosclerosis with increased IMT, carotid plaque and carotid atherosclerosis was higher in rural communities compared with urban communities (41.6% vs 30.8%) [35]. Similar results were reported from a mixed-population cardiovascular survey in Japan where a higher prevalence of increased IMT and carotid plaques was reported in rural populations compared with urban populations (27% vs. 24% and 46% vs. 37%, respectively) [32].

### 3.4. Prevalence of Carotid Artery Stenosis

The prevalence of carotid artery stenosis was reported in six studies and ranged from 0.4% to 16.1%. The lowest prevalence was reported in the China National Stroke Prevention Project [35] while the highest prevalence was reported in the targeted vascular screening program in rural Warren, Pennsylvania, USA [25]. The ultrasound assessment for carotid artery sclerosis varied by study. Details on the individual criteria are included in Table 3.

### 3.5. Risk Factors

Commonly addressed factors for atherosclerosis in our review were age, gender, dyslipidaemia, BMI, waist/hip ratio, physical activity, tobacco and alcohol use, diet, salt intake, education level, female menopausal status, depression, systolic blood pressure, medical and family history of diabetes, hypertension, cardiovascular disease and obesity. A range of biomarkers were analysed across the studies, including total cholesterol, low density lipid lipoprotein (LDL-C), high level density lipoprotein (HDL-C), triglycerides, fasting glucose, glycosylated hemoglobin (HbA1c), insulin levels and fatty acids.

The most significant risk factors associated with increased IMT were age [30,32], male gender [30], hypertension [30,32,33], smoking [30,32,33], total cholesterol level [30,33], high LDL-C [30,33], low HDL-C level [32,33,38], alcohol consumption [30], high fasting glucose levels [30], lower education levels [30] and diabetes [33]. Additionally, increased IMT was associated with a lower ankle-brachial index (ABI) [37], decreased cognitive performance [34], poor sleep quality [26], active snorers [40], and COPD and airflow limitation [36]. No association was found between increased IMT and low bone mineral density (BMD) [31], elevated homocysteine levels [24] or the presence of an earlobe crease [27].

Carotid plaque was associated with the following risk factors: advancing age [32,35,38,44], male gender [35,38,44], hypertension [32,35,38,44], smoking [32,35,38], alcohol consumption [35,38], high BMI [44], obesity [35], diabetes [32,35,38], dyslipidaemia [35], high LDL-C level [35], physical inactivity [35] and lower education level [41]. In addition, the presence of carotid plaque was higher in participants with COPD and airflow limitation [36] and in snorers [40]. No association was found between carotid plaque and elevated homocysteine levels [24] or low BMD [31]. Details on the statistically significant associated risk factors for increased IMT and carotid plaque can be found in the Supplementary Material, Tables S3 and S4.

Similarly, common risk factors for carotid atherosclerosis (encompassing increased IMT and carotid plaque) reported in the China National Stroke Prevention Project China National Stroke Prevention study were increasing age, male gender, rural residence, smoking, physical inactivity, obesity, hypertension, diabetes and dyslipidemia [35].

## 4. Discussion

To our knowledge, this is the first systematic review to examine the prevalence of carotid atherosclerosis and the utilization of carotid ultrasound screening on asymptomatic rural populations. We provided a comprehensive search strategy that included 22 articles in our review, capturing single and multiple reports from general population studies, to address not only the prevalence of carotid atherosclerosis but also the utilization of carotid ultrasound and different ultrasound determinants of carotid pathology in rural communities.

A large proportion of studies we reviewed were sub-studies conducted as a component of larger community multi-system health checks, population-based cohorts, or cross-sectional studies. Hence, there has been a lack of well-designed community-based vascular screening programs for carotid artery disease as a primary outcome measure. Ultrasound determinants for carotid atherosclerosis included increased IMT and/or the presence of carotid plaque and/or carotid artery stenosis. Assessment criteria for these determinants varied across studies. Interestingly, none of the studies included measures of local carotid stiffness, carotid plaque morphology or neovascularization.

The estimated prevalence of carotid plaque was available from studies conducted in three East Asian countries and estimated that carotid plaque was present in 34.1% of the rural asymptomatic population. Prevalence data for carotid stenosis were available from five studies across three countries and ranged from 0.4% to 16.1%. Data on the prevalence of increased IMT were available from five studies and ranged from 11.2% to 41.5%. Due to the differences in criteria for IMT and carotid artery stenosis assessment, we could not provide an overall estimation of prevalence.

With limited evidence, our review suggests a high prevalence rate of carotid atherosclerosis in rural populations residing primarily in East Asian countries. This result is somewhat comparable with results from systematic reviews and meta-analyses conducted in predominantly urban populations [23,48,49]. A recently published systematic review on the global prevalence of carotid plaque and carotid stenosis reported that, in 2020, 21% (816 million) and 1.5% (58 million) of people aged 30 to 79 lived with carotid plaque and carotid atherosclerosis, respectively [23]. Data from another systematic review and meta-analyses on moderate asymptomatic carotid stenosis showed a prevalence of 4.2% [48]. Interestingly, the results from two studies included in our review [32,35] showed higher prevalence of carotid atherosclerosis among participants living in rural areas as compared with their urban counterparts. These results align with the results from a comprehensive systematic review and meta-analysis of carotid atherosclerosis among Chinese adults, where a high burden of the disease (>70%) was attributed to rural living [49].

As shown in our results, age and male gender prevail as non-modifiable risk factors. Advancing age was associated with increased IMT or the presence of carotid plaque [30,32,35,38,44], suggesting the influence of aging in the pathogenesis of atherosclerosis as it leads to vascular structural changes that trigger increased local stiffness and impaired endothelial function [50,51]. In line with previous investigations [48,49], our results also show that carotid atherosclerosis is more prevalent in men than in women. Of clinical relevance, several potentially modifiable risk factors were also identified to be associated with carotid artery disease. Hypertension and smoking were common significant risk factors for increased IMT and carotid plaque reported in the reviewed studies. Additionally, few studies reported on physical activity, diabetes and alcohol consumption. Significant factors associated with carotid plaque were dyslipidaemia and increased LDL-C levels. The association between carotid atherosclerosis and these modifiable risk factors has been confirmed in previous epidemiological studies [52,53,54,55]. Furthermore, recent evidence suggests that over 70% of cardiovascular disease is attributable to modifiable risk factors, with hypertension being a significant contributor [56].

While current screening for asymptomatic atherosclerosis in the general population is controversial, recommendations against carotid screening rely predominantly on studies presenting the unproven benefits of preventive carotid interventions such as carotid endarterectomy and stenting [14]. However, atherosclerosis is a systemic disease, and its presence in one vascular bed can predict its presence in another [7,15,16,17]. Moreover, imaging markers such as pathological IMT, carotid plaque and carotid artery stenosis have been shown to be predictive of first time and recurrent vascular events [17,57,58,59]. The benefits of the early diagnosis of carotid atherosclerosis could improve both risk stratification and the detection and modification of potential risk factors associated with atherosclerosis. Indeed, our review demonstrated several modifiable risk factors associated with atherosclerosis plaque burden. Most of the rural population studies we reviewed did not examine all known risk factors. This also likely impacts the quality of evidence for guideline development for rural carotid ultrasound screening programs.

Our systematic review has several limitations. First, the main limitation of our review is that we estimated the overall prevalence of carotid plaque based on eight studies, all of which were conducted in East Asian countries. Despite the extensive search, we were unable to detect relevant studies from other regions, which limits our ability to generalize our results to other rural populations and races. Second, the low number of studies reporting on the prevalence of increased IMT and carotid artery stenosis as well as the differences in the criteria of these ultrasound markers, prevented us from providing further analyses and reporting. The criteria for IMT measurement cutoffs were inconsistent across the studies reviewed, varying from 0·9 mm to 1·8 mm. Only one study [24] defined the criteria in accordance with the Manheim IMT and carotid plaque consensus [47]. The criteria and cutoff points for low, moderate and severe stenosis varied based on the methods of measurement used, which were also not always reported. Lastly, there was insufficient data from multivariate analyses on the risk factors for IMT and carotid plaque to allow us to conduct robust meta-analyses. We were also unable to identify any significant risk factors associated with carotid stenosis due to insufficient data in the available studies.

In summary, our review examined studies published between 1999 and 2020 on rural carotid atherosclerosis using carotid ultrasound screening. With limited evidence, our findings demonstrate a significant burden of carotid atherosclerosis in rural populations mainly residing in East Asian countries. As these results are notably confined to a limited number of regions, there is insufficient evidence to generalize beyond the reviewed countries. We confirm the paucity of prevalence data and associated risk factors for carotid atherosclerosis in asymptomatic rural populations. Considering rural health inequalities, large-scale epidemiological studies in rural communities are required to determine the burden of carotid atherosclerosis or carotid stiffness (as an emerging biomarker of carotid atherosclerosis and global vascular risk) [60,61]. Finally, as many of the identified associated risk factors are modifiable, the detection of early carotid disease might reduce the burden of future cardio- and cerebrovascular disease.

## Figures and Tables

**Table 1:** Detailed characteristics of included studies (n = 22).

Authors	Year	Country	Setting	Sample Size (*n*)	Age Range	Men (%)	Ultrasound Determinants
Mannami et al. * [43]	1999	Japan	rural	249	60–74	100	Carotid artery stenosis
Kitamura, et al. * [32]	2004	Japan	rural/urban	1129	60–74	100	IMT, carotid plaque
Cui et al. [37]	2006	Japan	rural	732	60–79	100	IMT, carotid plaque
Galvao, et al. [25]	2010	USA	rural	758	>55	38	Carotid artery stenosis
Yano et al. [28]	2010	China	rural	618	≥45	41 42	IMT
Liang Y, et al. [33]	2014	China	rural	1499	>60	41.2	Carotid plaque
Lee et al. [40]	2014	Korea	rural	7330	≥40	39	IMT, carotid plaque
Zhan et al. ** [38]	2016	China	rural	3789	≥45	41.2	carotid plaque
Zhao et al. ** [39]	2016	China	rural	3789	≥45	41.2	carotid plaque
Li Y et al. [24]	2016	China	rural	2291	>55	44.34	IMT, carotid plaque, carotid artery stenosis
Kobayashi et al. [44]	2017	Japan	rural	309	>40	44.33	IMT, carotid plaque
Gao et al. ** [29]	2017	China	rural	3725	≥45	58	IMT
Guan et al. ** [30]	2017	China	rural	3509	≥45	41.6	IMT
Lou, et al. ** [41]	2017	China	rural	3789	≥45	41.2	carotid plaque
Kudo et al. [36]	2017	Japan	rural	2099	>40	42.4	carotid plaque
Jiang et al. [31]	2018	China	rural	756	55–65	44.16	IMT
Wang et al. [35]	2018	China	rural/urban	84,880	≥40	46.3	IMT, carotid plaque, carotid artery stenosis
Ren, et al. ** [45]	2018	China	rural	3789	≥45	41.2	IMT
Brutto, et al. *** [26]	2019	Ecuador	rural	561	≥40	42	IMT
Dong et al. ** [42]	2019	China	rural	3789	≥45	41.2	IMT, carotid plaque
Brutto et al. *** [27]	2019	Ecuador	rural	570	>40	42	IMT
Brutto et al. *** [34]	2020	Ecuador	rural	561	>40	42	IMT

IMT—intima media thickness, * report from the same investigation, ** report from the Tijanjin Brain Study, *** report from Atahulpa Project

**Table 2:** Prevalence of increased IMT from retrieved studies.

Study	Country	Sample Size	Prevalence IMT (%)	Ultrasound Criteria
Kitamura et al., 2004	Japan	817	25.8	≥1·1 mm
Liang et al., 2014	China	1499	11.2	≥1·8 mm
Li et al., 2016	China	2291	27.6	≥1 mm
Jiang et al., 2018	China	754	41.5	≥0·9 mm
Brutto et al., 2020 (Atahulpa Project)	Ecuador	561	25·0	≥0·9 mm

**Table 3:** Prevalence of carotid artery stenosis from retrieved studies.

Study	Country	Sample Size	Cases (n)	Prevalence Carotid Artery Stenosis (%)	Ultrasound Criteria	Criteria for Reporting Prevalence
Mannami et al.,1999	Japan	249	40	16.1% (11.7–21.2) *	<25% 25% to >50% >50%	from <25% to >50%
Galvao et al., 2010	USA	758	81	10.6%(8.5–13.1) *	Mild: (1%–39%) Moderate: (40%–59%) Severe: (60%–79%) Critical: (80%–99%) Occluded	from mild (1–39%) to occluded
Liang Y et al., 2014	China	1499	145	9.6% (8.2–11.2) *	Moderate ≥ 50% Severe ≥ 70%	>50%
Li Y et al., 2016	China	2291	132	5.8% (4.8–6.7) *	stenosis reported > 50%	>50%
Wang et al., 2018	China	42220	-	0.4% (0.4–0.5)	mild < 50%, moderate 50% to 69%, severe 70% to 99%, occlusion	>50%

* CI was not reported in the studies, the calculations were based on sample size and estimate prevalence.

## Data Availability

Not applicable.

## Supplementary Materials

The following are available online at https://www.mdpi.com/article/10.3390/jpm11090897/s1: Table S1. Search strategy for studies conducting ultrasound screening among rural population/Ovid MEDLINE/; Table S2. Quality assessment scores for risk of bias; Table S3. Risk factors for increased carotid Intima Media Thickness (IMT) from retrieved studies; Table S4. Risk factors for presence of carotid plaque from retrieved studies.
