# Supplementary material for: Carotid Ultrasound Screening Programs in Rural Communities: A Systematic Review
Source: J Pers Med. 2021 Sep 8;11(9):897. doi: 10.3390/jpm11090897 (PMC8465856; doi:10.3390/jpm11090897)
Supplement: Supplementary file 1 [file jpm-11-00897-s001.zip › jpm-1307891-supplementary.pdf]

Carotid Ultrasound Screening Programs in Rural Communities: A Systematic Review

Marjana Petrova [1,*], Hosen Kiat [1,2,3], Alex Gavino [1] and Craig S McLachlan [1]

[1] Centre for Healthy Futures, Health Faculty, Torrens University, Australia, 5/235 Pyrmont St, Pyrmont, NSW 2009, Australia; hosen.kiat@chi.org.au (H.K.) ; alex.gavino@torrens.edu.au (A.G.); craig.mclachlan@torrens.edu.au (C.S.M.)

[2] Faculty of Medicine, Health and Human Sciences, Macquarie University, Australia, Level 3, 75 Talavera Rd, Macquarie University, NSW, Sydney, 2109, Australia

[3] School of Rural Medicine, UNSW, Sydney Australia, 559 East Street, Albury, NSW 2640, Australia

*Correspondence: marjana.petrova@education.torrens.edu.au; Tel.: +61-401-595-535

Supplementary Material

Table. S1 Search strategy for studies conducting ultrasound screening among rural population/Ovid MEDLINE/

Table. S2 Quality assessment scores for risk of bias

Table. S3 Risk factors for increased carotid Intima Media Thickness (IMT) from retrieved studies

Table. S4 Risk factors for presence of carotid plaque from retrieved studies

Table. S1 Search strategy for studies conducting ultrasound screening among rural population/Ovid MEDLINE/

	**Ovid/ Medline**
1	carotid*.mp.
2	Carotid Artery,Common/or Carotid Arteries/or Carotid Intima-Media Thickness/or Carotid Stenosis/or Carotid Artery Disease
3	ultrasound*.mp.
4	sonograph*.mp
5	ultrasonograph*.mp
6	ultrasonograph*.mp. Or Ultrasonography/
7	rural*.mp
8	remote*.mp
9	regional*.mp.
10	Rural Health Services/or rural*mp or Hospitals, Rural/or Rural Population
11	1 or 2
12	3 or 4 or 5 or 6
13	7 or 8 or 9 or 10
14	11 and 12 and 13

Table. S2 Quality assessment scores for risk of bias

Quality Scores/based on STROBE/						
**Study**	**Sample population**	**sample size**	**participation**	**outcome assessment**	**Analytical methods**	**total score s**
Mannami et al.* [43]	2	1	2	2	2	9
Kitamura, et al.* [31]	1	1	0	2	2	6
Cui et al. [37]	2	1	0	2	2	7
Galvao et al. [24]	1	1	1	2	1	6
Yano et al. [27]	1	1	1	2	2	7
Liang Y et al. [33]	2	1	2	2	2	9
Lee et al. [40]	2	1	1	2	2	8
Zhan et al.**[38]	1	1	1	2	2	7
Zhao, et al.**[39]	1	1	1	2	2	7
Li Y et al. [32]	1	0	0	2	2	5
Kobayashi et al. [46]	1	1	1	2	2	7
Gao et al.**[28]	1	1	1	2	2	7
Guan et al.**[29]	1	1	1	1	2	6
Lou et al.**[41]	1	1	1	2	2	7
Kudo et al. [36]	2	1	0	2	2	7
Jiang et al. [30]	2	1	2	2	2	8
Wang et al. [35]	2	2	1	2	2	9
Ren et al.**[44]	1	1	1	2	2	7
Brutto et al.*** [25]	2	1	2	2	2	8
Dong et al.**[42]	1	1	1	2	2	7
Brutto et al.*** [26]	1	1	0	2	2	6
Brutto et al.*** [34]	2	1	2	2	2	8

** report from same investigation*

** report from Tijanjin Brain Study

*** report from Atahulpa Project

Quality assessment scale adapted from Song et al [23]

Low risk score = 2

Moderate risk score =1

High risk score 0

Max total score=10

Table. S3 Risk factors for increased carotid Intima Media Thickness (IMT) from retrieved studies*

Risk factor	Study	Country	OR	95% CI	p value
**Age**					
	Kitamura et al.,2004[31]	Japan/Northeast	1.04	1.00-1.80	0.02
	Guan et al., 2017[29]	China	1.06	1.05-1.07	<0.001
**Male gender**					
	Guan et al., 2017[29]	China	2.02	1.73-2.35	<0.001
**Hypertension**					
	Kitamura et al.,2004[31]	Japan/Southwest	1.09	1.20-3.10	0.005
	Kitamura et al.,2004[31]	Japan/Northeast	2.00	1.20-3.30	0.01
	Guan et al., 2017[29]	China	2.38	1.18-2.85	<0.001
	Liang et al., 2018[33]	China	2.45	1.52-3.95	-
**Smoking**					
	Liang et al., 201433	China	1.51	1.03-2.23	-
	Guan et al., 201729	China	1.60	1.36-1.89	<0.001
**Total cholesterol**					
	Kitamura et al., 2004[33]	Japan/Southwest	1.30	1.10-1.70	0.02
	Kitamura et al., 2004[33]	Japan/Northeast	1.40	1.10-1.90	0.008
	Guan et al., 2017[29]	China	1.09	0.02-0.17	0.01
**LDL-C**					
	Guan et al., 2017[29]	China	1.20	1.13-1.27	<0.001
	Liang et al., 2014[33]	China	1.47	1.01-2.12	-
**Alcohol consumption**	Guan et al., 2017[29]	China	1.44	1.13-1.75	<0.001
**HDL-C**					
	Kitamura et al., 2004[31]	Japan/Southwest	0.60	0.50–0.90	0.002
**Fasting glucose ≥6.0mmol/l**					
	Guan et al., 2017[29]	China	1.49	1.20-1.85	<0.001
**Education**					
	Guan et al., 2017[29]	China	0.84	0.82-0.96	<0.001
**Diabetes**					
	Kitamura et al.,2004[31]	Japan/Northeast	2.50	1.20-5.20	0.02
	Liang et al., 2014[33]	China	1.50	1.05-2.15	-

* Only statistically significant risk factors were reported in this table (OR, 95% CI, p<0.05)

Table. S4 Risk factors for presence of carotid plaque from retrieved studies*

Risk factor	Study	Country	OR	95% CI	p value
**Age**					
	Kitamura et al., 2004 [31]	Japan/Northeast	1.8	1.4-2.2	<0.001
	Wang et al.,2018[35]	China	7.33**	6.31-8.42	<0.001
	Zhan et al., 2016[38]	China	1.07	1.05-1.08	<0.001
	Kobayashi et al., 2019[46]	Japan	1.27	1.06-1.32	0.05
**Male gender**					
	Wang et al.,2018[35]	China	1.21	1.11-1.31	<0.001
	Zhan et al., 2016[38]	China	1.69	1.35-2.11	<0.001
	Kobayashi et al., 2019[46]	Japan	2.48	1.428-5.02	<0.01
**Hypertension**					
	Kitamura et al., 2004[31]	Japan/Southwest	1.90	1.2-3.1	0.005
	Kitamura et al., 2004[31]	Japan/Northeast	2.10	1.3-3.2	0.002
	Wang et al., 2018[35]	China	1.62	1.53-1.72	<0.001
	Zhan et al., 2016[38]	China	1.41	1.16-1.71	<0.001
**Smoking**					
	Wang et al., 2018[35]	China	1.70	1.52-1.90	<0.001
	Zhan et al., 2016[38]	China	1.45	1.11-1.88	0.006
**Alcohol consumption**					
	Zhan et al., 2016[38]	China	0.57	0.35-0.94	0.026
**BMI**					
	Kobayashi et al., 2019[46]	Japan	1.32	1.24-2.74	<0.01
**Obesity**					
	Wang et al., 2018[35]	China	1.23	1.11-1.36	<0.001
**Diabetes**					
	Zhan et al., 2016[38]	China	1.47	1.17-1.86	0.001
	Wang et al., 2018[35]	China	1.46	1.37-1.56	<0.001
**Dyslipidemia**					
	Wang et al., 2018[38]	China	1.48	1.36-1.62	<0.001
**LDL-C**	Zhan et al., 2016[35]	China	3.92	2.70-5.69	<0.001
**Physical Inactivity**					
	Wang et al., 2018[35]	China	1.34	1.24-1.44	<0.001

* Only statistically significant risk factors were reported in this table (OR, 95% CI, p<0.05)

** for age ≥70 years
